# The Novel New Jersey Eyewitness Instruction Induces Skepticism but Not Sensitivity

**DOI:** 10.1371/journal.pone.0142695

**Published:** 2015-12-09

**Authors:** Athan P. Papailiou, David V. Yokum, Christopher T. Robertson

**Affiliations:** 1 James E. Rogers College of Law, University of Arizona, Tucson, Arizona, United States of America; 2 Department of Psychology, University of Arizona, Tucson, Arizona, United States of America; University of Rome, ITALY

## Abstract

In recent decades, social scientists have shown that the reliability of eyewitness identifications is much worse than laypersons tend to believe. Although courts have only recently begun to react to this evidence, the New Jersey judiciary has reformed its jury instructions to notify jurors about the frailties of human memory, the potential for lineup administrators to nudge witnesses towards suspects that they police have already identified, and the advantages of alternative lineup procedures, including blinding of the administrator. This experiment tested the efficacy of New Jersey’s jury instruction. In a 2×2 between-subjects design, mock jurors (*N* = 335) watched a 35-minute murder trial, wherein identification quality was either “weak” or “strong” and either the New Jersey or a “standard” instruction was delivered. Jurors were more than twice as likely to convict when the standard instruction was used (OR = 2.55; 95% CI = 1.37–4.89, *p* < 0.001). The New Jersey instruction, however, did *not* improve juror's ability to discern quality; rather, jurors receiving those instructions indiscriminatingly discounted “weak” and “strong” testimony in equal measure.

## Introduction

The legal framework for evaluating the admissibility of eyewitness identification evidence was first laid out by the United States Supreme Court in the 1970s. [1, 2] A catalog of empirical studies has since proven that admissible eyewitness identifications are surprisingly misleading to jurors, especially when extracted from suggestive lineup procedures. [3] As the list of defendants wrongfully convicted due to eyewitness testimony continues to grow, the legal system has begun searching for a reform solution. One category of reform is to revisit the admissibility rules to more aggressively police what testimony reaches the jury. A second category of reform instead focuses on jurors as consumers of the (already admitted) testimony. We aim to assist jurors distinguish between trustworthy and unreliable testimony by teaching them about the foibles of human memory, especially when tainted by unduly suggestive lineup procedures. As advocates of this strategy have noted, “we now have enough empirical evidence… to insist that jurors should be informed about the proneness to error of whatever [identification] procedure is used.” [4]

The New Jersey judiciary has recently led the second reform category when it release a new judicial instruction in July 2012—an instruction carefully constructed to inform lay jurors of the state-of-the-science on eyewitness memory and how to leverage that knowledge in assessing such testimony. [5] The instruction admonishes jurors that “[e]yewitness identification evidence must be scrutinized carefully” because “research has shown that there are risks of making mistaken identifications.” [6] It proceeds to dispel the belief that memory is “like a video recording”, explaining that—in layperson terms—the memory process is composed of acquisition, retention, and retrieval stages. The instruction proceeds to address “specific factors that [the juror] should consider”, generally pertaining to “the observations and perceptions on which the identification was based, the witness’s ability to make those observations and perceive events, and the circumstances under which the identification was made.” Guidance is given on those factors, such as that “a witness's level of confidence, standing alone, may not indicate the reliability of the identification”, and that an officer administering a lineup, if not blinded as to who is the suspect, “may intentionally or unintentionally convey that knowledge to the witness.” These factors are further discussed in the Methodology section. The full instruction on eyewitness testimony can be found in Appendix A.

The New Jersey instruction has received considerable attention. Distinguished psychologists in the areas of memory and eyewitness testimony have applauded the New Jersey instruction in that it “relie[s] on, and receives strong support from, decades of research from cognitive psychology.” [7] New Jersey Supreme Court Chief Justice Stuart Rabner noted that “[t]he instructions are designed to minimize the risk of wrongful convictions and help jurors reach informed, just decisions.” [5] A press release from the Innocence Project commended the instruction as “the first in the nation that explain[s] the way memory works and the factors that can affect the reliability of eyewitness identifications.” [8] Director Barry Scheck proclaimed that “these instructions will revolutionize the way that juries scrutinize identification evidence.” [8] An ad-hoc committee of the National Academy of Sciences approved of how the New Jersey instruction directed judges to focus on factors relevant to the specific case.

We present an empirical question, namely: Does administration of an enhanced judicial instruction, such as the one promulgated by New Jersey, actually affect, and ideally improve the sensitivity, of jury decision-making? Our randomized controlled experiment indicates that the new instructions might not be as efficacious as hoped.

### A Uniquely New Instruction

To our knowledge, no prior empirical study has tested the New Jersey instruction. Past attempts to improve the efficacy of other textual instructions on eyewitness testimony have been largely unsuccessful. A notable example is the *Telfaire* instruction, which was adopted during the 1970s and is now the most commonly given cautionary instruction. [9] This instruction reminds jurors to consider whether the witness had the capacity and opportunity to observe the offender, and to consider whether the identification was a result of the witness’s own recollection, along with other general factors for credibility including consistency of multiple identifications. Several experimental tests have shown that the *Telfaire* instruction fails to improve sensitivity to the quality of the eyewitness testimony. A grab-bag of mixed results have instead found everything else: no effect [10], across-the-board skepticism [11, 12], and perhaps even across-the-board credulity.

The first experiment by Greene [11] used a 2×2 between-subjects design with a videotaped mock criminal trial, where the identification was either strong or weak and the *Telfaire* instruction either was or was not provided. The instruction pushed guilty rates to the floor, reducing guilty verdicts from 41% to 7% in the strong condition, and pressing flat at 3% across the weak conditions. Functionally, that instruction actually *desensitized* jurors, rendering them skeptical of the otherwise strong testimony. A second experiment found that a revised *Telfaire* instruction—one with simplified, reorganized language—again induced skepticism without any sensitivity gains. Although work by Cutler, Dexter, & Penrod failed to replicate a skepticism effect, they nonetheless observed that “[t]he *Telfaire* instruction proved completely ineffective at sensitizing jurors to eyewitness evidence.” [10] A review by Penrod & Cutler lamented that “judges' instructions do not serve as an effective safeguard against mistaken identifications and conviction—they may produce a slight skepticism effect, but they appear to reduce sensitivity to witnessing and identification conditions.” [9]

The New Jersey instruction notably differs from the *Telfaire* instruction by providing substantially more detail about memory mechanisms and how eyewitness identification evidence can be misleading. Such additional instruction has been found effective, albeit in a different pedagogical format. Pawlenko, Safer, Wise, & Holfeld [13], for example, tested whether a 24-slide PowerPoint presentation (which they called the “interview-identification-eyewitness factor” teaching aid, or the “I-I-Eye aid” for short), which explained various factors affecting the reliability of eyewitness identifications, affected verdict rates amongst mock jurors (*N* = 293 undergraduate students) reading a vignette of a criminal murder trial. They found a promising result, namely, that the I-I-Eye aid increased sensitivity to the strength of identification procedures, relative to two different standard instructions. When standard instructions were read, guilty verdict rates floated around 30% regardless of whether the eyewitness testimony was weak or strong. Conversely, when the I-I-Eye aid provided, guilty verdict rates were 55% or 16%, respectively, depending on if the testimony was strong or weak—a significant increase in sensitivity.

Despite the promising result found by Pawlenko and colleagues, it might be difficult to implement in a real court setting. For example, it is unclear how the I-I-Eye aid would actually be delivered (i.e., by the judge or via expert testimony). Courts might be more likely to implement a more traditional textual instruction, read aloud to the jury, than to undertake the role of classroom instructor and PowerPoint presenter. Further research is required into the efficacy of enhanced instruction as presented verbally from a judge.

Although our study’s purpose bears similarity to Pawlenko et al.’s study, we are ultimately concerned with the New Jersey instruction on its own merits, as an exemplar of how read-aloud instructions from the judge might be revised to more effectively teach about the foibles of eyewitness testimony. And it is at this empirical nexus that the New Jersey instruction holds out its promise. Scholars have speculated that “it might be necessary to educate jurors about the fallibility of identification witnesses in more detail.” [14] The New Jersey instruction aims to do precisely that. Will the enhanced instruction mimic the efficacy of the I-I-Eye aid, by improving sensitivity, or will it instead continue to flounder like the *Telfaire* instruction? This experiment provides data to begin answering that question.

## Methods

### Ethics Statement

This research was approved as exempt because it used standard survey techniques.

University of Arizona

Human Subjects Protection Program

IORG0000177

IRB00000291

### Design and Stimuli

In a 2×2 between-subjects factorial design, participants acted as mock jurors by watching one of four videos of a criminal trial, systematically varied (through video editing) by the strength of eyewitness testimony (“ID Quality”: weak or strong) and type of judicial instruction used (“Instruction”: standard or enhanced). The video, although abbreviated (it lasted from 30–40 minutes depending on condition), contained the basic elements of a trial: opening statements from both the prosecution and defense; direct and cross-examination of three witnesses; closing arguments; and jury instructions read aloud by the judge.

The trial, *State of New Jersey v*. *Peter Brown*, involved allegations that the defendant robbed and murdered the cashier of a gas station convenience store. The case hinged on the testimony of a witness who was present when the crime happened, and who identified the defendant as the culprit during a photographic lineup.

Video editing was used to manipulate the strength of this eyewitness testimony, according to several of the criteria delineated as important within the novel New Jersey instruction (and by extension the body of empirical research on eyewitness testimony). For example, in the “strong” ID Quality condition, the police officer administering the lineup was blinded as to which lineup participant was the suspect, the witness was instructed that the suspect may or may not be in the lineup, and the lineup included eight photographs. In the “weak” ID Quality condition, the officer was aware of who was the suspect, failed to instruct that the suspect may be absent, and used only five photographs. Table 1 provides a full list of the differences across ID Quality conditions. All other aspects of the testimony—and indeed all other evidence—were held constant across conditions.

**Table 1:** Operationalization of ID Quality.

Factor	Weak	Strong
Did the interviewing officer ask the eyewitness about media exposure?	No	Yes
Was the eyewitness instructed to avoid discussing the crime and avoid the media?	No	Yes
Did the interviewing officer ask leading questions about the appearance of the perpetrator?	Yes	No
Did the interviewing officer ask leading questions about the quality of the witness's view of the perpetrator?	Yes	No
Were standardized identification procedure instructions used?	No	Yes
How many photos were in the lineup array?	5	8
Did the description of the suspect match the appearance of line-up participants?	No	Yes
Was the interviewing officer unaware of which lineup participant was the suspect?	No	Yes
Was the witness instructed that the perpetrator may or may not be in the lineup?	No	Yes
Did the interviewing officer provide confirmatory feedback immediately after the identification?	Yes	No

Columns indicate differences in the key witness's testimony across the “weak” and “strong” ID Quality conditions. All other aspects of the evidence were held constant across conditions.

With this operationalization of ID Quality, jurors should—if they are accurately assessing the quality of eyewitness testimony—be significantly more likely to convict the defendant in the “strong” condition than in the “weak” condition. Indeed, the two ID Quality conditions were deliberately crafted to embody obviously different indicia of eyewitness testimony quality, thereby providing fertile ground for the New Jersey instruction to do its salutary work, if in fact it does so work.

The Instruction variable was manipulated by having the judge read either a “standard” instruction, modeled after a minimal eyewitness instruction commonly used in Florida, or the novel instruction recently promulgated by the New Jersey courts (*New Jersey Eyewitness Instruction*, 2012), which we label as the “enhanced” instruction for convenience. Florida was selected, as opposed to the older New Jersey instruction, to assess how the “enhanced” instruction stacks up against other language currently in use. This was, for example, the instruction used in the recent, high-profile Trayvon Martin case. Moreover, the Florida instruction is relatively sparse, containing general questions to consider, such as “Did the witness seem to have an accurate memory” or “Did the witness seem to have an opportunity to see and know the things about which the witness testified?” It does not teach about the frailty of human memory or the potentially biasing impact of unduly suggestive lineup procedures. In other words, it lacks precisely the sort of empirically informed facts that the novel New Jersey instruction sought to warn about. Thus, if the unique instructional elements of the New Jersey instruction have a salutary impact on how juries assess eyewitness testimony, then it should be especially notable in comparison to the “standard” instruction.

Crossing the ID Quality and Instruction variables affords an opportunity to test two hypotheses related to the New Jersey instruction. First, does it have any effect on jury decision-making? A finding that jurors are less (or more) likely to find the defendant guilty when the “enhanced” instruction is used relative to the “standard” instruction would provide an affirmative answer. Second, and importantly, if there is an effect, is it an effect that increases diagnostic accuracy, that is, sensitivity to eyewitness testimony quality? This would be revealed by an interaction effect: ideally, the use of the “enhanced” instruction should result in lower conviction rates when the ID Quality is “low” rather than “high.” After all, if the “enhanced” instruction similarly decreased convictions in the “high” ID Quality condition, then it would obtain a decrease in false positives, but only at the cost of an increase in false negatives. P To fully test this possibility, the case facts (across ID Quality conditions; see Table 1) were deliberately manipulated to either avoid or succumb to risks explicitly laid out in the “enhanced” (but not “standard”) instructions. For example, the lineups in the “weak” and “strong” conditions entailed five and eight photos, respectively, while the “enhanced” instruction directs that “[a] minimum of six persons or photos should be included.” (The “standard” instruction is silent on the issue). Thus, a juror following the “enhanced” instruction should discount the testimony in the “weak” condition, but not in the “strong” condition.

### Participants

The study population was drawn from Amazon Mechanical Turk, with an advertisement to participate in an approximately one-hour research experiment in exchange for $2.00 payment. Five hundred and four persons proceeded past the informed consent page, although 143 cases (28%) were removed for failure to complete the task (most left during the video), and another 26 cases (5%) were removed for one of two quality-assurance reasons: (a) entry of “garbage” text (e.g., smashed keys or copied-and-pasted question prompts); or (b) completing the experiment in less than 30 minutes, an impossibly fast time (the video stimuli lasted at least 30 minutes). The final sample (*N* = 335) was predominantly white (80%), tended to be female (57%), and was about 35 years old. Most (83%) had at least some college credit.

### Procedure and Measured Variables

Participants completed the experiment online, via a survey programmed and hosted on Qualtrics. The survey flow entailed an initial informed consent page, a demographics questionnaire, and then an instruction page including the admonition to “please treat this case with the same seriousness and diligence that you would use in a real trial.” The experiment itself followed: participants watched one of the four mock trial videos, and then answered a series of questions about their verdict preferences and use of the evidence.

The primary dependent variable of interest, answered immediately after the video, was a binary response (“Yes, guilty” or “No, not guilty”) to the question, “Based on the evidence and the instructions provided by the judge, has the prosecutor proved, beyond a reasonable doubt, that the Defendant is guilty?” A follow-up question asked for a brief written description of the reasons for their decision.

Additional questions probed issues of judicial instruction comprehension, confidence in verdict, use of evidence, and the reliability of the eyewitness testimony. Responses were indicated on 6-point Likert scales. Specifically, participants were asked to assess the following (with Likert anchors in parentheses): “I understand the Judge's instructions” (“strongly disagree” to “strongly agree”); “How confident are you in your verdict?” (“not at all confident” to “absolutely confident”); and “Barbara Dunn's eyewitness testimony was influential in your verdict decision” (“strongly disagree” to “strongly agree”).

## Results

### Verdict

**Fig 1:**
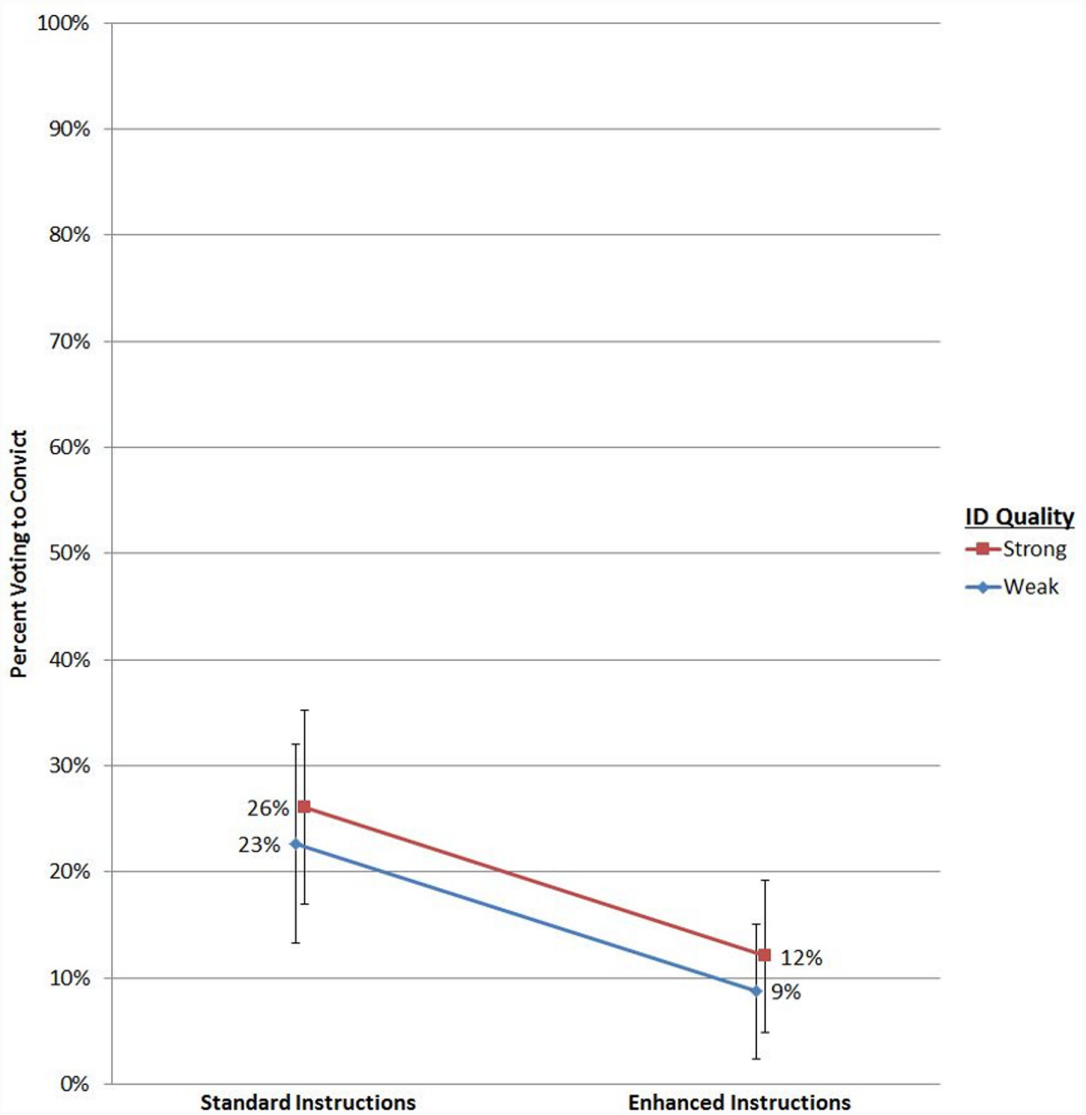
Proportion of Guilty verdicts (and 95% confidence intervals) by Instruction and ID Quality.

Fig 1 shows the proportions of guilty verdicts by experimental condition. The “enhanced” instruction reduced the number of guilty verdicts, although it appears to do so about equally for both levels of ID Quality. In the “standard” Instruction condition, conviction rates are similar in the “weak” (23%) and “strong” (26%) ID Quality conditions, and remain similar—albeit substantially reduced—when the “enhanced” instruction is used (weak = 9%; strong = 12%).

Binary logistic regression was used to statistically assess the impact of ID Quality and Instruction (and their interaction) on the likelihood of a guilty verdict, as well as provide additional control (beyond randomization) of demographic covariates. Table 2 displays the results of three regression models. The first model regressed verdict (0 = Not Guilty; 1 = Guilty) on the independent variables using dummy coding: Instruction_Standard (0 = enhanced; 1 = standard) and ID Quality_Weak (0 = strong; 1 = weak). Model two builds upon model one, by adding the possible Instruction by ID Quality interaction term. Model three includes demographic covariates of Male (0 = female; 1 = male), Minority (0 = white; 1 = non-white), College (0 = less than Bachelor's degree; 1 = Bachelor's degree or higher), and Age_10 (age at 10 year intervals); because the interaction term was non-significant even with demographic covariates, and its inclusion complicates interpretation of the other coefficients, model three does not include it.

**Table 2:** Logistic regressions predicting a verdict of guilt.

	Model 1	Model 2	Model 3
	ß (S.E.)	ß (S.E.)	ß (S.E.)
Intercept	-2.04 (.29)	-1.99 (.33)	-2.59 (.55)
Instruction_Standard	1.02 (.31)**	0.95 (.42)*	0.94 (.32)**
ID Quality_Weak	-0.24 (.31)	-0.69 (.49)	-0.22 (.29)
Instruction_Standard × ID Quality_Weak	-	0.17 (.63)	-
Male	-	-	-0.13 (.31)
Minority	-	-	0.65 (.35)°
College	-	-	0.72 (.32)*
Age_10	-	-	0.02 (.12)
Null deviance (df)	311.86 (334)	311.86 (334)	311.86 (334)
Residual deviance (df)	299.54 (332)	299.47 (311)	290.19 (328)

Null and residual deviances are shown at the bottom of the table, for assessing overall model fit.

° *p <* 0.10.

* *p* < 0.05.

** *p* < 0.01.

The statistical results confirm what visual inspection of Fig 1 suggests. Referring to model three (which is the model of best fit, as revealed be the reduction in residual deviance; see Table 2), there is a substantial main effect of Instruction: use of the “standard” instruction relative to the “enhanced” instruction more than doubles the odds that the defendant will be found guilty (odds ratio = 2.55; 95% CI = 1.37–4.89; *p* < 0.001). ID Quality, on the other hand, does *not* have a significant main effect on verdict (*p* = 0.44). There is also no evidence of an interaction effect (*p* = 0.84). The interaction is not significant in model two nor a full model (such as model three) containing demographic covariates; the *p* = 0.84 value is from the full model. To summarize it differently, use of the novel New Jersey instruction substantially reduced the likelihood that the defendant would be found guilty, but its reducing effect was the same regardless of whether the eyewitness identification testimony was weak or strong.

### Self-Perceived Comprehension, Confidence, and Influence

Table 3 summarizes the means and standard deviations of the Likert-scale responses. Analysis of Variance (ANOVA) was used to assess whether any main effects of Instruction or ID Quality, or the interaction of the two, were statistically significant.

**Table 3:** Participant Comprehension, confidence, and influence.

ID Quality	Instruction	*n*	Comprehension of Instruction	Confidence in Verdict	Influence of Testimony
Strong	Enhanced	83	5.51 (0.85)	4.61 (1.17)	4.12 (1.38)
Strong	Standard	88	5.61 (0.56)	4.77 (1.13)	4.43 (1.31)
Weak	Enhanced	80	5.56 (0.61)	4.66 (1.08)	4.15 (1.34)
Weak	Standard	84	5.61 (0.62)	4.65 (1.19)	4.40 (1.14)

Means and standard deviations of mock jurors' comprehension of the judicial instruction, confidence in their verdict, and agreement that the testimony of the key eyewitness was “influential in [their] verdict decision.” All responses were on a 1–6 point Likert scale, with higher numbers indicating greater comprehension, confidence, or agreement.

Self-perceived comprehension of judicial instructions was not significantly affected by either ID Quality (*p* = 0.74) or Instruction (*p* = 0.29), and no interaction was found (*p* = 0.67). Responses instead hovered around 5.55 across conditions, indicating that most subjects “somewhat agree[d]” or “agree[d]” that they understood the judge's instructions, regardless of instruction type.

Confidence in verdict was likewise unaffected by ID Quality (*p* = 0.76), Instruction (*p* = 0.54), or the interaction of the two (*p* = 0.51). These scores were all within the 4.6–4.7 range, indicating most jurors were “confident” in their verdicts.

There was, however, an effect of Instruction on self-assessments of whether the testimony of the key eyewitness influenced participants' verdicts. In particular, mock jurors who received the “enhanced” instruction were significantly more likely to believe that the eyewitness testimony did *not* influence their verdict (*F*(1, 331) = 4.00, *p* = 0.046). This was true in both the “strong” and “weak” ID Quality conditions, where estimates of influence were about 0.30 points (on the 6-point Likert scale) lower when the “enhanced” instruction was used (specifically, 4.12 versus 4.43 in the “strong” condition, and 4.15 versus 4.40 in the “weak” condition). Lastly, the effect of ID Quality was again non-significant (*p* = 0.99), as was the interaction term (*p* = 0.84).

## Discussion

This study tested the efficacy of New Jersey’s approach to ensuring that jurors have decreased reliance on faulty eyewitness testimony and increased reliance on trustworthy testimony. We did so by providing jurors with an “enhanced” judicial instruction, that cautions against undue reliance on imperfect human memory, especially in the presence of suggestive lineup procedures. After a careful review of the empirical evidence on eyewitness testimony, the New Jersey judiciary crafted precisely such an instruction.

Unfortunately, our experimental data provides a mixed review of the efficacy of the New Jersey instruction. On the one hand, the use of the New Jersey instruction substantially reduced juror reliance on weak identification evidence, as compared to the Florida-based instruction. This suggests that jurors can listen to the judge's admonitions and they take into consideration that guidance. This is an especially promising result when considered against the backdrop of empirical work which has found that jurors are unaffected by such instructions. Nietzel, McCarthy, & Kerr [15] for example, in a meta-analysis of 150 relevant effect sizes, find only a weak correlation between conditions of instruction and no-instruction and effect on juror behavior (mean *r* = 0.07).

When jurors fail to follow instructions, it is often because they do not understand what is being asked of them. [16] Here, as evidenced by the high ratings of self-perceived comprehension of judicial instructions (most jurors at least “somewhat agree[d]” they understood), it seems they did not find themselves so baffled. Of course, the fact that jurors self-report understanding does not necessarily mean they are not confused. The review by Devine [16] notes the “disconcerting finding” that, despite poor performance on comprehension tests, “jurors [nonetheless] typically report understanding the judge's instructions with no problems.” Nonetheless, our between-subjects design shows that the instruction is impactful in the direction that would be consistent with understanding (a decreased rate of conviction).

On the other hand, the New Jersey instruction also equally reduced juror reliance on *strong* identification evidence. The failure to find an interaction of the enhanced instruction with the quality of the eyewitness testimony contradicts the hypothesis that the New Jersey instruction increases sensitivity, improving the abilities of jurors to discern the difference between a strong and a weak identification. Instead, when jurors are confronted with a catalog of the foibles of human memory and the extra risks posed by unduly suggestive lineup procedures, they indiscriminatingly discount any and all eyewitness identification testimony.

This result is suboptimal: the New Jersey instructions will likely increase the rate at which guilty defendant are exonerated (i.e., a false negative). Yet it might still be an improvement over the “standard” instruction, at least if one agrees with Blackstone's argument that reducing false positives is more important than reducing false negatives (“better that ten guilty persons escape than that one innocent suffer”).

It appears that jurors are swayed by the content of the instruction (the empirically-informed details about how to scrutinize eyewitness testimony) and *want* to apply this knowledge to the case at hand, they cannot, for whatever reason, do so accurately. Perhaps, for instance, our mock jurors knew they should discount testimony whenever a certain criterion was met, but were unsure at what threshold that criterion should be deemed satisfied. The “enhanced” instruction is often silent as to such issues of magnitude. For example, it states that “high levels of stress can reduce an eyewitness's ability to recall and make an accurate identification”, but says nothing about what constitutes a “high” level. As one more example, it notes that the “amount of time any eyewitness observes an event may affect the reliability of an accurate identification”, but provides little guidance on the duration-to-reliability function, except for noting a difference between “brief or fleeting contact” and “more prolonged exposure.” A juror unsure of whether a threshold has been met might, as the law instructs (in terms of reasonable doubt), err on the side of doubt and presume the testimony, even if otherwise “strong”, surpasses the threshold. This could explain why the enhanced disclosure reduces reliance on even the “strong” testimony. On the other hand, the “weak” versus “strong” conditions used here were (deliberately) operationalized along bright-line criteria (e.g., either the interviewing officer was blinded or not; either standardized instructions were used or not; either six or more participants were used in the lineup or not). Thus, an inability to apply fuzzy criteria seems at best a partial explanation.

An alternative explanation is that the mere *length* of the “enhanced” instruction drove the observed effect, which cannot be ruled out by our findings. The “standard” instruction took about 5 minutes to read, whereas the “enhanced” instruction took about 15 minutes to read. This three-fold increase in hearing about the problems of eyewitness testimony could make the issue more salient and, therefore, more of a concern in general, whatever the case facts may be. The design of our experiment cannot distinguish this possibility from a failure to accurately apply the criteria (a limitation of internal validity). But the New Jersey instruction, relative to other extant instructions, simply *is* that much longer. Thus, for purposes of external validity, and in particular for appreciating the likely effect of the New Jersey instruction, the ultimate effect of interest might be the same regardless of underlying reason: jurors are substantially less likely to convict on the basis on eyewitness testimony. On the other hand, a real trial would be substantially longer than that of our mock trial stimuli. Perhaps the effect of instruction length is dependent on what *proportion* of the total trial experience it occupies. In our study, 5 and 15 minutes of instruction in an approximately 35 minute trial constitutes 14% and 43% of the total trial—a substantial difference. In a 25-hour trial, in contrast, the proportions would only constitute 0.3% and 1.0%, a difference that is likely to be lost relative to the bulk of the trial experience. Nonetheless, future experiments should tease apart the role of length versus ability to apply the guidelines, the results of which would be useful for improving judicial instructions.

An alternative explanation is that the observed skepticism reflects more about source credibility (and by extension the depth of cognitive processing) than the New Jersey instruction’s effectiveness. In particular, jurors might interpret the judge’s decision to read the enhanced instruction—one which belabors the inaccuracies of eyewitness testimony, without much counterbalanced discussion of its conditions of accuracy—as an implicit signal that said testimony should, in fact, not be trusted. Why else, a juror might wonder, would the judge bother to make such a fuss about it?

A related dynamic has been described by Cutler et al. [10] found that adversarial expert testimony induced greater sensitivity but that non-adversarial expert testimony (e.g., a court appointed expert) resulted in a skepticism effect with no sensitization. They proposed an explanation in terms of the elaboration likelihood model (ELM) of persuasion. [17] The ELM predicts that people (not unreasonably) process and scrutinize information more critically when the source of that information is perceived as less credible *a priori*. In contrast, a credible source’s statements are more likely to be accepted at face value. The link for present purposes is that a juror, who trusts the judge and misinterprets the judge’s lengthy instruction as an implicit signal the testimony is faulty, might fail to engage in the increased cognitive elaboration required to apply the instruction to the facts at hand. Easier instead to simply discount the testimony wholesale, without any elaboration.

A source credibility explanation could also accommodate the fact that the I-I-Eye Aid used by Pawlenko et al. [13] induced sensitivity. Specifically, the I-I-Eye Aid was *not* presented within the context of the mock trial. Study participants rather viewed the PowerPoint slides before engaging the trial materials, in a manner suggesting it was the researchers, not the judge, providing the guidance. This is not a context wherein subjects would misinterpret the guidance as an implicit signal from a court.

The present study is not designed to test source credibility effects. The relevant test would be to have the content of the New Jersey instruction delivered by someone other than the judge. Who that might be in practice is a complicated issue, but methodologically in the laboratory, one could simply pull the instruction outside the context of the mock trial, similar to how Pawlenko et al. [13] presented the I-I-Eye Aid; the prediction being that in such case the instruction would induce sensitivity. Likewise, one would predict that the I-I-Eye Aid, if presented by the judge rather than researchers, could induce skepticism rather than sensitivity.

As a mock jury study with an online study population, the usual cautions apply. The case was not a full-length trial with jurors coerced to appear and then allowed to deliberate collectively on the real consequences for a defendant to redress real injuries for a victim. This experiment was hypothetical, used a sample that was not precisely representative of the jury pool, and so forth. Nonetheless, this experiment was a randomized, controlled trial with several hundred jury eligible adults. And it used a 30–40 minute trial stimulus of relatively high ecological validity, in terms of providing a video capture of all key trial elements, and verbatim use of the New Jersey instruction.

The lackluster performance of the New Jersey instruction should signal that the task of reforming judicial instructions on eyewitness testimony is not complete. Our results provide a warning to judges to be wary of the possibility that enhanced instructions will lead jurors to overly discount reliable testimony. Our results also motivate the need for further research on how judicial instructions on eyewitness testimony can be best crafted, and how they can be best delivered.

Nonetheless, the New Jersey instructions could be used as a means to increasing the diagnosticity of criminal trials. Even if the “enhanced” instruction caused jurors to indiscriminately discount testimony, it might still be leveraged—by way of a more active judicial role. In particular, one suggestion is to allow judges discretion as to when to apply the instruction, and as to which parts of it to read, so that only the weak elements of an identification are criticized. In this way, the judge might increase the sensitivity of the jury, as the ultimate consumer of the instruction.

Some of this is already done, in obvious fashion: if there was no lineup conducted, then obviously there is no need to give cautionary instructions pertaining to a lineup procedure. But a more nuanced approach would be to allow the judge to decline to read the lineup instruction, even if one was performed, so long as the used procedures met certain gold-standard criteria. For instance, if the lineup entailed more than six photographs, the presiding officer was blinded as to who was the suspect, and so forth, then the judge could elect to simply not read the warning. In terms of our experiment, this would be akin to reading the instruction in the “weak” condition but not reading the instruction in the “strong” condition. This could avoid unduly alarming the jurors in the latter scenario, and thereby avoid the increased rate of false negatives that we observed experimentally.

Of course, this suggestion does raise its own problems, neither of which are trivial. The first arises from tasking judges with deciding when the “gold-standard” has been met. Can they do this accurately? This is another empirical question, but there is reason to believe that they may have higher capacity than jurors to resolve this particular predicate question.

## Appendix A: New Jersey (“Enhanced”) Jury Instructions

### Pertaining to Eyewitness Testimony

Mr. Peter Brown, as part of his general denial of guilt, contends that the State has not presented sufficient reliable evidence to establish beyond a reasonable doubt that he is the person who committed the alleged offenses. The burden of proving the identity of the person who committed the crime is upon the State. For you to find this defendant guilty, the State must prove beyond a reasonable doubt that this defendant is the person who committed the crime. The defendant has neither the burden nor the duty to show that the crime, if committed, was committed by someone else, or to prove the identity of that other person. You must determine, therefore, not only whether the State has proven each and every element of the offenses charged beyond a reasonable doubt, but also whether the State has proven beyond a reasonable doubt that this defendant is the person who committed it.

The State has presented the testimony of Mrs. Barbara Dunn. You will recall that this witness identified the defendant in court as the person who committed the crime of armed robbery. The State also presented testimony that on a prior occasion before this trial, this witness identified the defendant as the person who committed these offenses. According to the witness, her identification of the defendant was based upon the observations and perceptions that she made of the perpetrator at the time these offenses was being committed. It is your function to determine whether the witness’s identification of the defendant is reliable and believable, or whether it is based on a mistake or for any reason is not worthy of belief. You must decide whether it is sufficiently reliable evidence that this defendant is the person who committed these offenses charged.

Eyewitness identification evidence must be scrutinized carefully. Human beings have the ability to recognize other people from past experiences and to identify them at a later time, but research has shown that there are risks of making mistaken identifications. That research has focused on the nature of memory and the factors that affect the reliability of eyewitness identifications.

Human memory is not foolproof. Research has revealed that human memory is not like a video recording that a witness need only replay to remember what happened. Memory is far more complex. The process of remembering consists of three stages: acquisition—the perception of the original event; retention—the period of time that passes between the event and the eventual recollection of a piece of information; and retrieval—the stage during which a person recalls stored information. At each of these stages, memory can be affected by a variety of factors.

Relying on some of the research that has been done, I will instruct you on specific factors you should consider in this case in determining whether the eyewitness identification evidence is reliable. In evaluating this identification, you should consider the observations and perceptions on which the identification was based, the witness’s ability to make those observations and perceive events, and the circumstances under which the identification was made. Although nothing may appear more convincing than a witness’s categorical identification of a perpetrator, you must critically analyze such testimony. Such identifications, even if made in good faith, may be mistaken. Therefore, when analyzing such testimony, be advised that a witness’s level of confidence, standing alone, may not be an indication of the reliability of the identification.

If you determine that the out-of-court identification is not reliable, you may still consider the witness’s in-court identification of the defendant if you find that it resulted from the witness’s observations or perceptions of the perpetrator during the commission of these offenses, and that the identification is reliable. If you find that the in-court identification is the product of an impression gained at the out-of-court identification procedure, it should be afforded no weight. The ultimate question of the reliability of both the in-court and out-of-court identifications is for you to decide.

To decide whether the identification testimony is sufficiently reliable evidence to conclude that this defendant is the person who committed these offenses charged, you should evaluate the testimony of the witness in light of the factors for considering credibility that I have already explained to you. In addition, you should consider the following factors that are related to the witness, the alleged perpetrator, and the criminal incident itself. In particular, you should consider:

1. The Witness’s Opportunity to View and Degree of Attention: In evaluating the reliability of the identification, you should assess the witness’s opportunity to view the person who committed these offenses at the time of these offenses and the witness’s degree of attention to the perpetrator at the time of these offenses. In making this assessment you should consider the following:
  - Stress: Even under the best viewing conditions, high levels of stress can reduce an eyewitness’s ability to recall and make an accurate identification. Therefore, you should consider a witness’s level of stress and whether that stress, if any, distracted the witness or made it harder for him or her to identify the perpetrator.
  - Duration: The amount of time an eyewitness has to observe an event may affect the reliability of an identification. Although there is no minimum time required to make an accurate identification, a brief or fleeting contact is less likely to produce an accurate identification than a more prolonged exposure to the perpetrator. In addition, time estimates given by witnesses may not always be accurate because witnesses tend to think events lasted longer than they actually did.
  - Weapon Focus: You should consider whether the witness saw a weapon during the incident and the duration of the crime. The presence of a weapon can distract the witness and take the witness’s attention away from the perpetrator's face. As a result, the presence of a visible weapon may reduce the reliability of a subsequent identification if the crime is of short duration. In considering this factor, you should take into account the duration of the crime because the longer the event, the more time the witness may have to adapt to the presence of the weapon and focus on other details.
  - Distance: A person is easier to identify when close by. The greater the distance between an eyewitness and a perpetrator, the higher the risk of a mistaken identification. In addition, a witness’s estimate of how far he or she was from the perpetrator may not always be accurate because people tend to have difficulty estimating distances.
  - Lighting: Inadequate lighting can reduce the reliability of an identification. You should consider the lighting conditions present at the time of the alleged crime in this case.
  - Disguises/Changed Appearance: The perpetrator’s use of a disguise can affect a witness’s ability both to remember and identify the perpetrator. Disguises like hats, sunglasses, or masks can reduce the accuracy of an identification. Similarly, if facial features are altered between the time of the event and a later identification procedure, the accuracy of the identification may decrease.
2. Prior Description of Perpetrator: Another factor for your consideration is the accuracy of any description the witness gave after observing the incident and before identifying the perpetrator. Facts that may be relevant to this factor include whether the prior description matched the photo or person picked out later, whether the prior description provided details or was just general in nature, and whether the witness's testimony at trial was consistent with, or different from, her prior description of the perpetrator.
3. Confidence and Accuracy: You heard testimony that Mrs. Barbara Dunn made a statement at the time she identified the defendant from a photo line-up concerning her level of certainty that the photograph she selected is in fact the person who committed the crime. As I explained earlier, a witness’s level of confidence, standing alone, may not be an indication of the reliability of the identification. Although some research has found that highly confident witnesses are more likely to make accurate identifications, eyewitness confidence is generally an unreliable indicator of accuracy.
4. Time Elapsed: Memories fade with time. As a result, delays between the commission of a crime and the time an identification is made can affect the reliability of the identification. In other words, the more time that passes, the greater the possibility that a witness’s memory of a perpetrator will weaken.
  In evaluating the reliability of a witness’s identification, you should also consider the circumstances under which any out-of-court identification was made, and whether it was the result of a suggestive procedure. In that regard, you may consider everything that was done or said by law enforcement to the witness during the identification process. You should consider the following factors:
  - Line-up Composition: A suspect should not stand out from other members of the lineup. The reason is simple: an array of look-alikes forces witnesses to examine their memory. In addition, a biased lineup may inflate a witness’s confidence in the identification because the selection process seemed so easy to the witness. It is, of course, for you to determine whether the composition of the lineup had any effect on the reliability of the identification.
  - Fillers: Lineups should include a number of possible choices for the witness, commonly referred to as “fillers.” The greater the number of choices, the more likely the procedure will serve as a reliable test of the witness’s memory. A minimum of six persons or photos should be included in the lineup.
  - Multiple Viewings: When a witness views the same person in more than one identification procedure, it can be difficult to know whether a later identification comes from the witness’s memory of the actual, original event or of an earlier identification procedure. As a result, if a witness views an innocent suspect in multiple identification procedures, the risk of mistaken identification is increased. You may consider whether the witness viewed the suspect multiple times during the identification process and, if so, whether that affected the reliability of the identification.

In determining the reliability of the identification, you should also consider whether the identification procedure was properly conducted.

1. Double-blind: A lineup administrator who knows which person or photo in the lineup is the suspect may intentionally or unintentionally convey that knowledge to the witness. That increases the chance that the witness will identify the suspect, even if the suspect is innocent. For that reason, whenever feasible, live lineups and photo arrays should be conducted by an officer who does not know the identity of the suspect. If a police officer who does not know the suspect’s identity is not available, then the officer should not see the photos as the witness looks at them. In this case, it is alleged that the person who presented the lineup knew the identity of the suspect. It is also alleged that the police did/did not compensate for that by conducting a procedure in which the officer did not see the photos as the witness looked at them. You may consider this factor when you consider the circumstances under which the identification was made, and when you evaluate the overall reliability of the identification.
2. Instructions: You should consider what was or what was not said to the witness prior to viewing a photo array. Identification procedures should begin with instructions to the witness that the perpetrator may or may not be in the array and that the witness should not feel compelled to make an identification. The failure to give this instruction can increase the risk of misidentification. If you find that the police [did/did not] give this instruction to the witness, you may take this factor into account when evaluating the identification evidence.
3. Feedback: Feedback occurs when police officers, or witnesses to an event who are not law enforcement officials, signal to eyewitnesses that they correctly identified the suspect. That confirmation may reduce doubt and engender or produce a false sense of confidence in a witness. Feedback may also falsely enhance a witness’s recollection of the quality of his or her view of an event. It is for you to determine whether or not a witness’s recollection in this case was affected by feedback or whether the recollection instead reflects the witness’s accurate perception of the event.

You may consider whether the witness was exposed to opinions, descriptions, or identifications given by other witnesses, to photographs or newspaper accounts, or to any other information or influence, that may have affected the independence of his/her identification. Such information can affect the independent nature and reliability of a witness’s identification and inflate the witness’s confidence in the identification.

You are also free to consider any other factor based on the evidence or lack of evidence in the case that you consider relevant to your determination whether the identifications were reliable. Keep in mind that the presence of any single factor or combination of factors, however, is not an indication that a particular witness is incorrect. Instead, you may consider the factors that I have discussed as you assess all of the circumstances of the case, including all of the testimony and documentary evidence, in determining whether a particular identification made by a witness is accurate and thus worthy of your consideration as you decide whether the State has met its burden to prove identification beyond a reasonable doubt. If you determine that the in-court or out-of-court identifications resulted from the witness's observations or perceptions of the perpetrator during the commission of these offenses, you may consider that evidence and decide how much weight to give it. If you instead decide that the identification is the product of an impression gained at the in-court and/or out-of-court identification procedures, the identifications should be afforded no weight. The ultimate issue of the trustworthiness of an identification is for you to decide.

If, after consideration of all of the evidence, you determine that the State has not proven beyond a reasonable doubt that Mr. Peter Brown was the person who committed these offenses, then you must find him not guilty. If, on the other hand, after consideration of all of the evidence, you are convinced beyond a reasonable doubt that Mr. Peter Brown was correctly identified, you will then consider whether the State has proven each and every element of these offenses charged beyond a reasonable doubt.

## Appendix B: Florida-Based (“Standard”) Jury Instructions

### Pertaining to Eyewitness Testimony

It is up to you to decide what evidence is reliable. Some things you should consider are: Did the witness seem to have an opportunity to see and know the things about which the witness testified? Did the witness seem to have an accurate memory? Was the witness honest and straightforward in answering the attorneys’ questions? Did the witness have some interest in how the case should be decided? A juror may believe or disbelieve all of or any part of the evidence or testimony of any witness.

## Appendix C: Additional Jury Instructions, Common to both “Standard” and “Enhanced” Conditions

Members of the Jury, thank you for your attention. Please listen to the instructions I am about to give you.

Mr. Peter Brown, the Defendant in this case, is accused of first degree felony murder of David Aims. If you have a reasonable doubt as to the guilt of the Defendant you should find the Defendant not guilty. If you have no reasonable doubt you should find the Defendant guilty. It is the evidence introduced at this trial and to it alone that you are to look for that proof. It is up to you to decide what evidence is reliable.

Before you can find the Defendant guilty of the first-degree felony murder, the state must prove the following three elements beyond a reasonable doubt: Number one, David Aims is dead. Number two, did this occur as a consequence of, and while Peter Brown was engaged in the commission of a robbery? Number three, Peter Brown was the person who actually killed David Aims. An issue in this case is whether the Defendant was present when the crime allegedly was committed. If you have a reasonable doubt that the Defendant was present at the scene of the alleged crime, it is your duty to find the Defendant not guilty. Finally, the decision to testify is the 5th Amendment right of the Defendant. The fact that the Defendant in this case did not testify should have no bearing on your verdict.

## Supporting Information

S1 Dataset Minimal dataset. This raw data file underlies our statistical analysis.
(CSV)

## References

[1] Neil v. Biggers, 409 U.S. 188 (1972).

[2] Manson v. Braithwaite, 432 U.S. 98 (1977).

[3] Nadel L, Sinnott-Armstrong WP. Memory and Law Oxford Series in Neuroscience, Law, and Philosophy. 1st Ed. Oxford University Press; 2012.

[4] Laudan L. Eyewitness Identifications One More Lesson on the Costs of Excluding Relevant Evidence. Perspectives on Psychological Science. 2012; 7(3): 272–274. 10.1177/1745691612443065 26168464

[5] New Jersey Courts. Supreme Court Releases Eyewitness Identification Criteria for Criminal Cases. 19 July 2012. Available: http://www.judiciary.state.nj.us/pressrel/2012/pr120719a.htm

[6] New Jersey Eyewitness Instruction. New Jersey Model Criminal Jury Charges. 2012. Available: http://www.judiciary.state.nj.us/pressrel/2012/jury_instruction.pdf

[7] Schacter DL, Loftus EF. Memory and law: what can cognitive neuroscience contribute? Nature Neuroscience. 2013; 16(2): 119–123. 10.1038/nn.3294 23354384

[8] Innocence Project. New Jersey Supreme Court issues new jury instructions that will greatly improve the way courts handle identification evidence. 19 July 2012. Available: http://www.innocenceproject.org/Content/New_Jersey_Supreme_Court_Issues_New_Jury_Instructions_That_Will_Greatly_Improve_the_Way_Courts_Handle_Identification_Evidence.php#

[9] Penrod S, Cutler B. Witness confidence and witness accuracy: Assessing their forensic relation. Psychology, Public Policy, and Law. 1995; 1(4): 817–845. 10.1037/1076-8971.1.4.817

[10] Cutler BL, Dexter HR, Penrod SD. Nonadversarial Methods for Sensitizing Jurors to Eyewitness Evidence. Journal of Applied Social Psychology. 1990; 20(14):1197–1207. 10.1111/j.1559-1816.1990.tb00400.x

[11] Greene E. Judge’s Instruction on Eyewitness Testimony: Evaluation and Revision. Journal of Applied Social Psychology. 1988; 18(3): 252–276.

[12] Ramirez G, Zemba D, Geiselman R. Judges' cautionary instructions on eyewitness testimony. American Journal of Forensic Psychology. 1996; 14(1): 31–66.

[13] Pawlenko NB, Safer MA, Wise RA, Holfeld B. A Teaching Aid for Improving Jurors’ Assessments of Eyewitness Accuracy. Applied Cognitive Psychology. 2013; 27(2), 190–197. 10.1002/acp.2895

[14] Bornstein BH, Hamm JA. Jury Instructions on Witness Identification. Court Review. 2012; 48(1/2): 48–53.

[15] Nietzel MT, McCarthy DM, Kerr MJ. Juries: The Current State of the Empirical Literature. Psychology and Law: The State of the Discipline. Perspectives in Law and Psychology. 1999; 1:10.

[16] Devine DJ. Jury decision making: The state of the science. NYU Press; 2012 Available: http://books.google.com/books?hl=en&lr=&id=jfTEAAJuFjEC&oi=fnd&pg=PP2&dq=JURY+DECISION+MAKING:+THE+STATE+OF+THE+SCIENCE&ots=bVYvvXVBl9&sig=oPJ2DMdfsErCWMrKypc7D9Lrsm8

[17] Petty RE, Caciopp JT. Communication and persuasion: Central and peripheral routes to attitude change. 1986. Retrieved from http://library.wur.nl/WebQuery/clc/535074

